# Supplementary material for: Toward a BT.2020 green emitter through a combined multiple resonance effect and multi-lock strategy
Source: Nat Commun. 2022 Aug 19;13:4876. doi: 10.1038/s41467-022-32607-3 (PMC9391368; doi:10.1038/s41467-022-32607-3)
Supplement: Supplementary file 1 — Supplementary Information [file 41467_2022_32607_MOESM1_ESM.pdf]

Supplementary Information: Toward a BT.2020 green emitter through a combined multiple resonance effect and multi-lock strategy

Junyuan Liu[1], Yunhui Zhu[2]*, Taiju Tsuboi[1], Chao Deng[2], Weiwei Lou[2], Dan Wang[1], Tiangeng Liu[1], Qisheng Zhang[1,3]*

[1] MOE Key Laboratory of Macromolecular Synthesis and Functionalization, Department of Polymer Science and Engineering, Zhejiang University, Hangzhou 310027, P.R. China.

[2] Zhejiang Hongwu Technology Co., Ltd., Taizhou 317100, P.R. China.

[3] State Key Laboratory of Clean Energy Utilization, Zhejiang University, Hangzhou 310027, P.R. China.

Email: zhuyunhui@hwoled.com, qishengzhang@zju.edu.cn

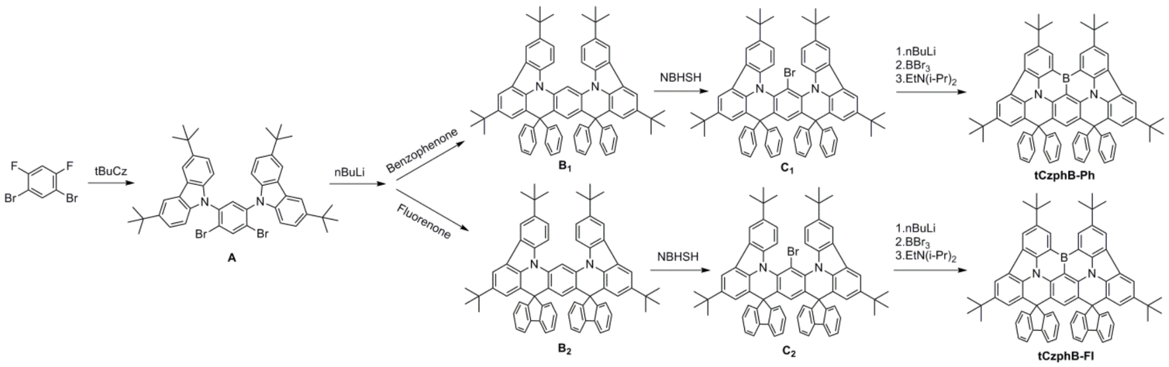

Supplementary Fig. 1 Synthetic route of tCzphB-Ph and tCzphB-Fl

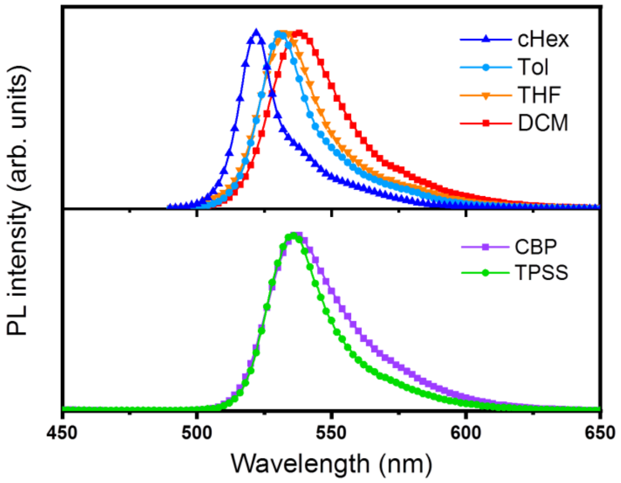

Supplementary Fig. 2 Normalized fluorescence spectra of tCzphB-Fl in different solutions (cyclohexane, toluene, tetrahydrofuran and dichloromethane) of 1×10^{-5} M and with CBP and TPSS as host at room temperature (RT).

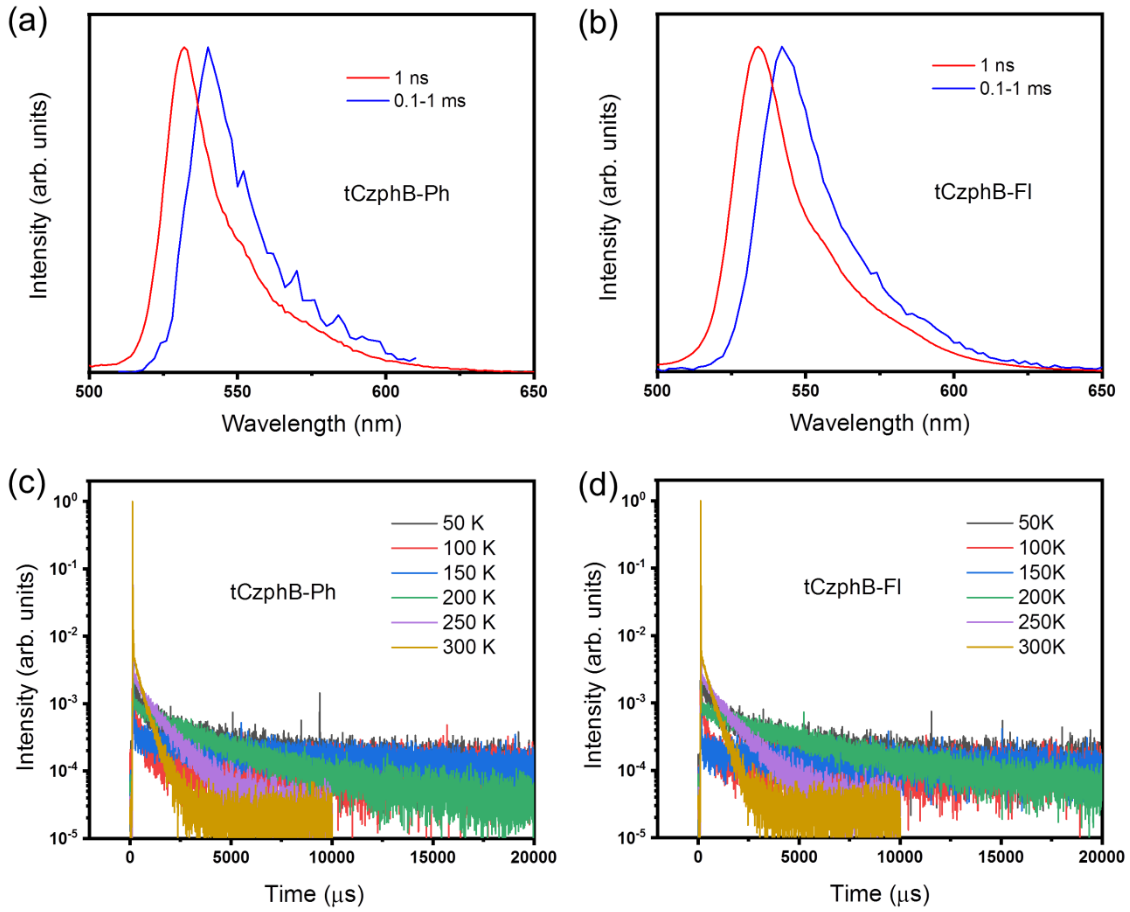

Supplementary Fig. 3 Emission and transient decay spectra. Fluorescence (1 ns component) and phosphorescence (0.2−1 ms component) spectra of tCzphB-Ph (**a**) and tCzphB-Fl (**b**) doped into the TPSS films (2 wt%) at 50 K. Transient PL decay spectra of tCzphB-Ph (**c**) and tCzphB-Fl (**d**) doped into the TPSS films (2 wt%) at 50−300 K.

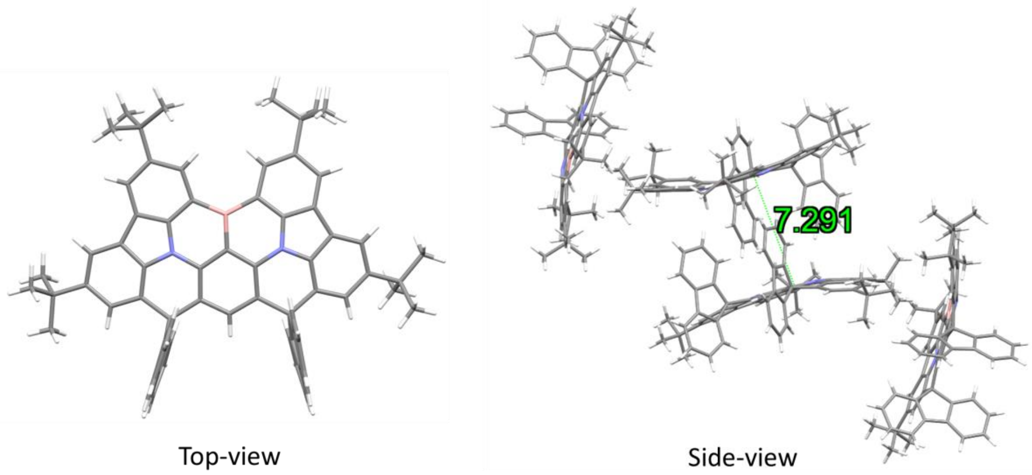

Supplementary Fig. 4 Crystal structure of tCzphB-Fl from top-view and side-view.

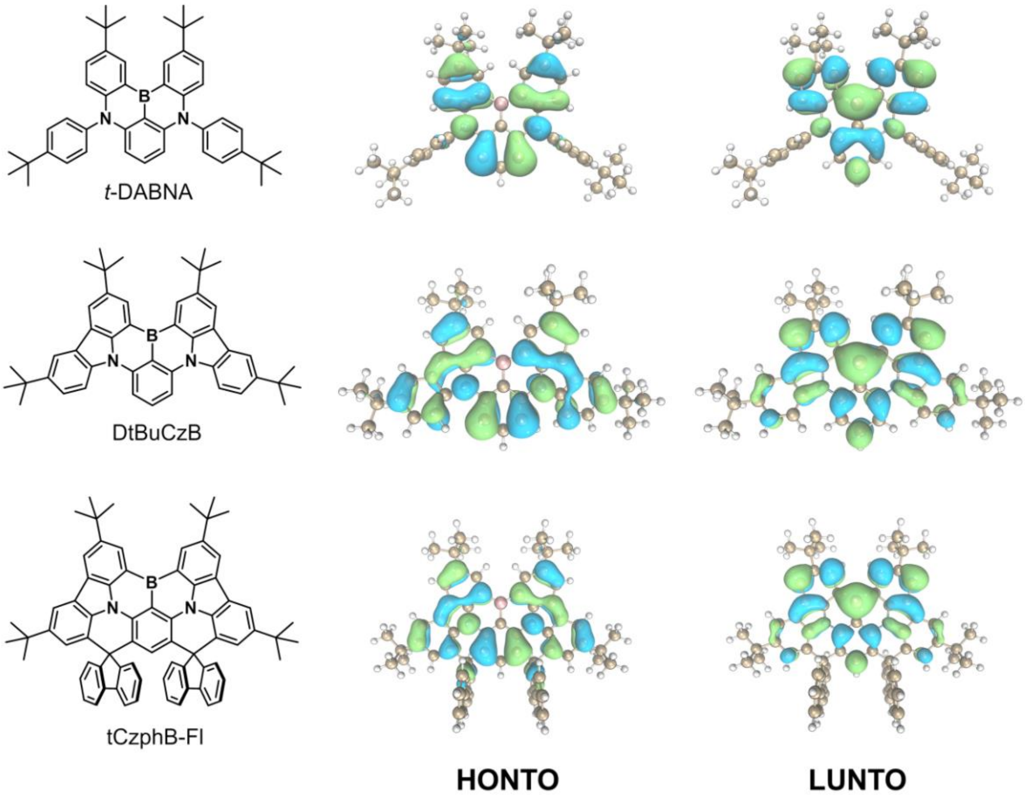

Supplementary Fig. 5 The natural transition orbitals of $S_1 \rightarrow S_0$ transition of *t*-DABNA, DtBuCzB and tCzphB-Fl. The orbital iso-surface distribution plots at their S_1 states calculated at the PBE0/6−31G (d, p) level.

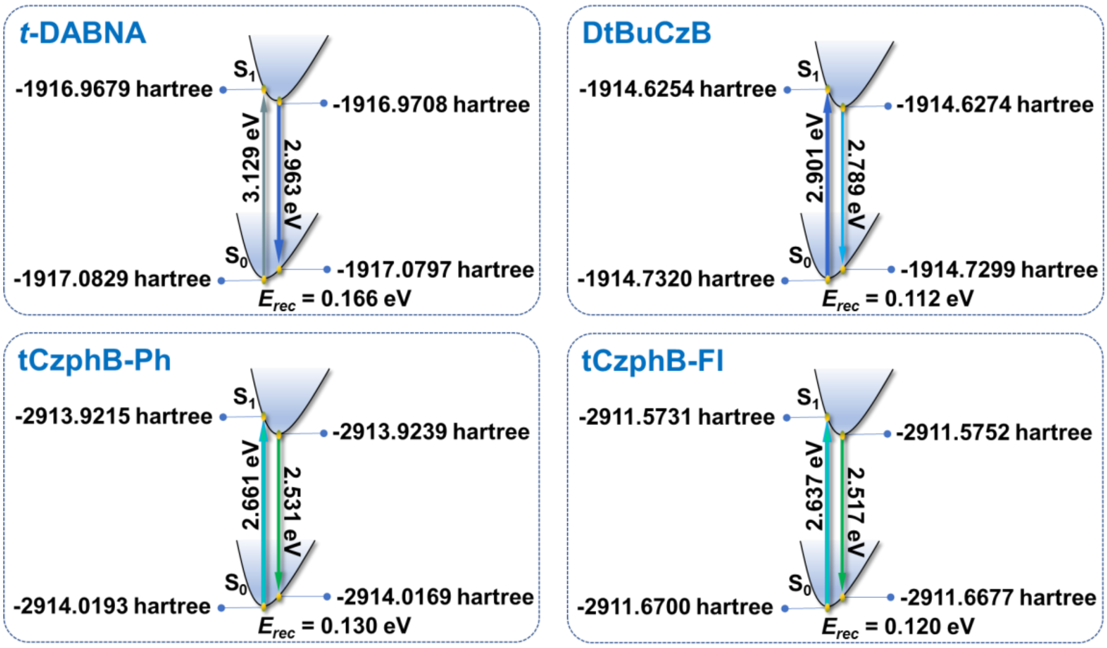

Supplementary Fig. 6 Potential energy surfaces in the ground and excited states. The transition and reorganization energies were calculated by TD-DFT at the PBE0/6-31G (d, p) level in toluene.

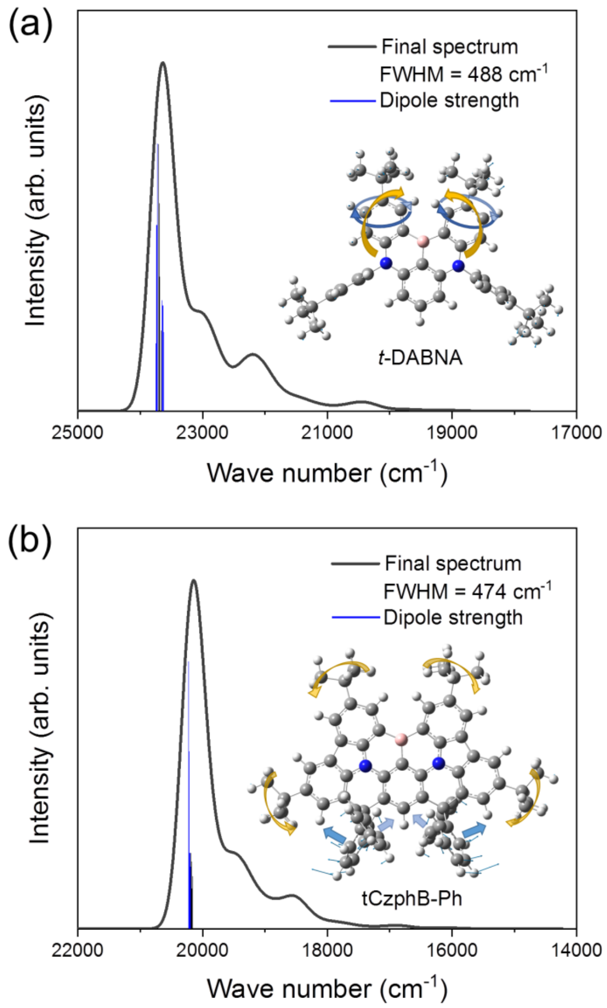

Supplementary Fig. 7 Spectral simulation. Simulated vibrationally-resolved fluorescence spectra with dipole strengths of different vibrational modes for **a** *t*-DABNA and **b** tCzphB-Ph.

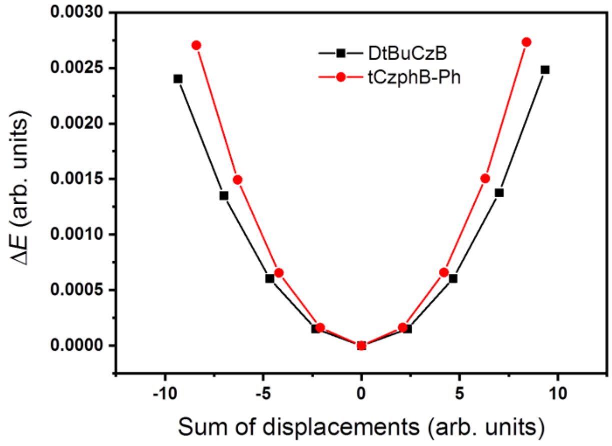

Supplementary Fig. 8 Vibrational potential energy curves of DtBuCzB and tCzphB-Ph on the basis of their major vibrational mode. Comparing the deformed geometric structures with the ground-state structure in Cartesian coordinates, the sum-modulus-difference for all atoms is recorded as a relative displacement.

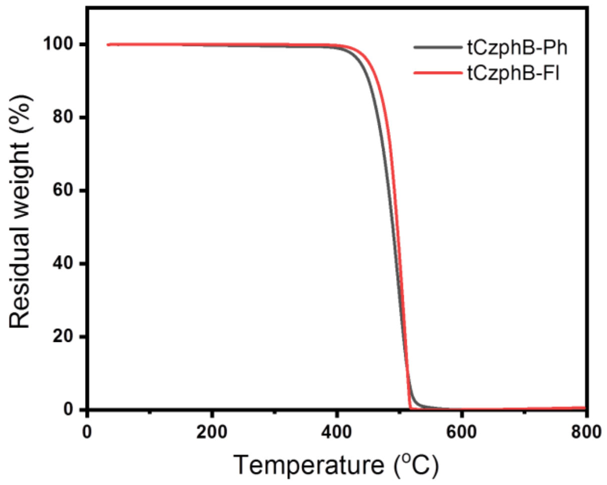

Supplementary Fig. 9 TGA Curves of investigated compounds under nitrogen flow. The 5 wt% weight-loss-temperatures for tCzphB-Ph and tCzphB-Fl are 437 ℃ and 450 ℃, respectively.

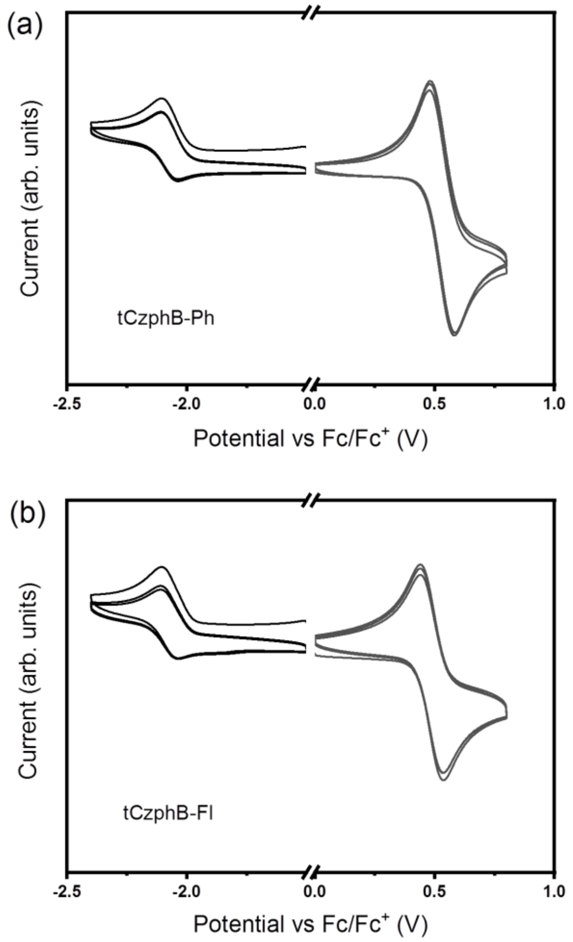

Supplementary Fig. 10 Cyclic voltammetry measurements. Cyclic voltammograms of **a** tCzphB-Ph and **b** tCzphB-Fl in DCM (oxidation) and DMF (reduction). The LUMO and HOMO energy level can be obtained from the reduction and oxidation curves following the formula: HOMO = -[E_{ox}-E(Fc/Fc^{+}) + 4.8] eV and LUMO = -[E_{re} – E(Fc/Fc^{+}) + 4.8] eV, respectively.

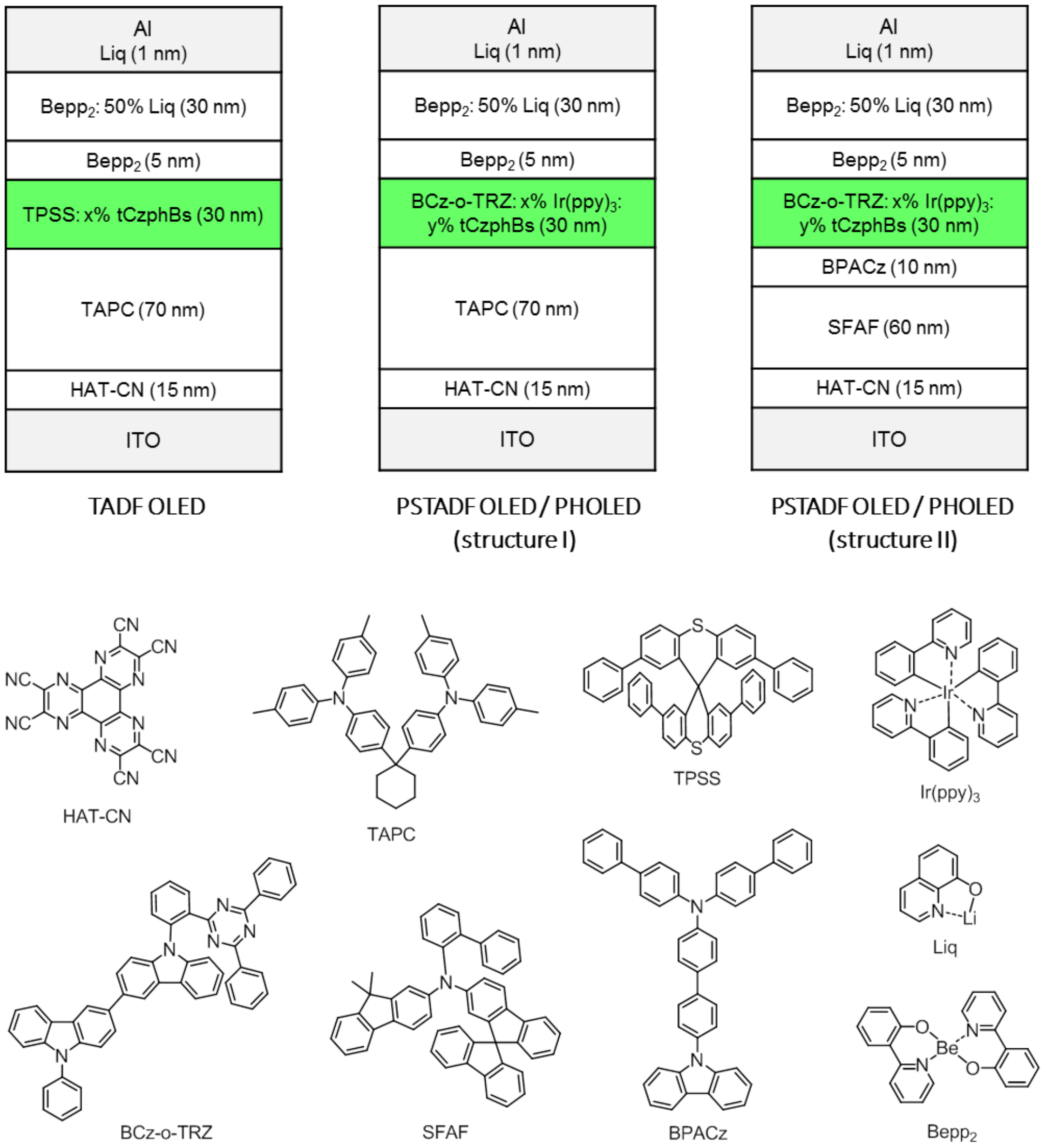

Supplementary Fig. 11 Structures of the investigated OLEDs and the molecular structures of the materials used in them.

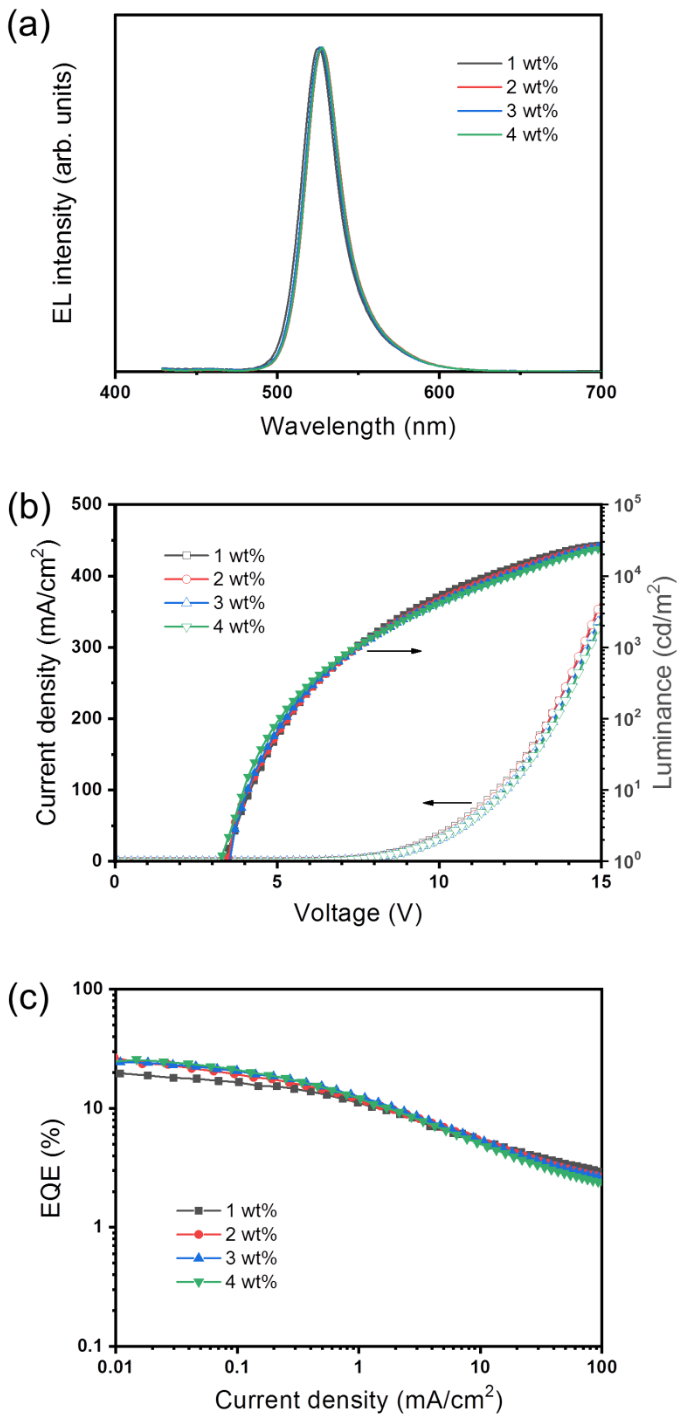

Supplementary Fig. 12 OLED characteristics. Electroluminescence spectra at 10 mA/cm^2 (**a**), current density–voltage–luminance characteristics (**b**) and EQE–current density characteristics (**c**) of the TADF OLEDs with different concentrations of tCzphB-Ph in TPSS.

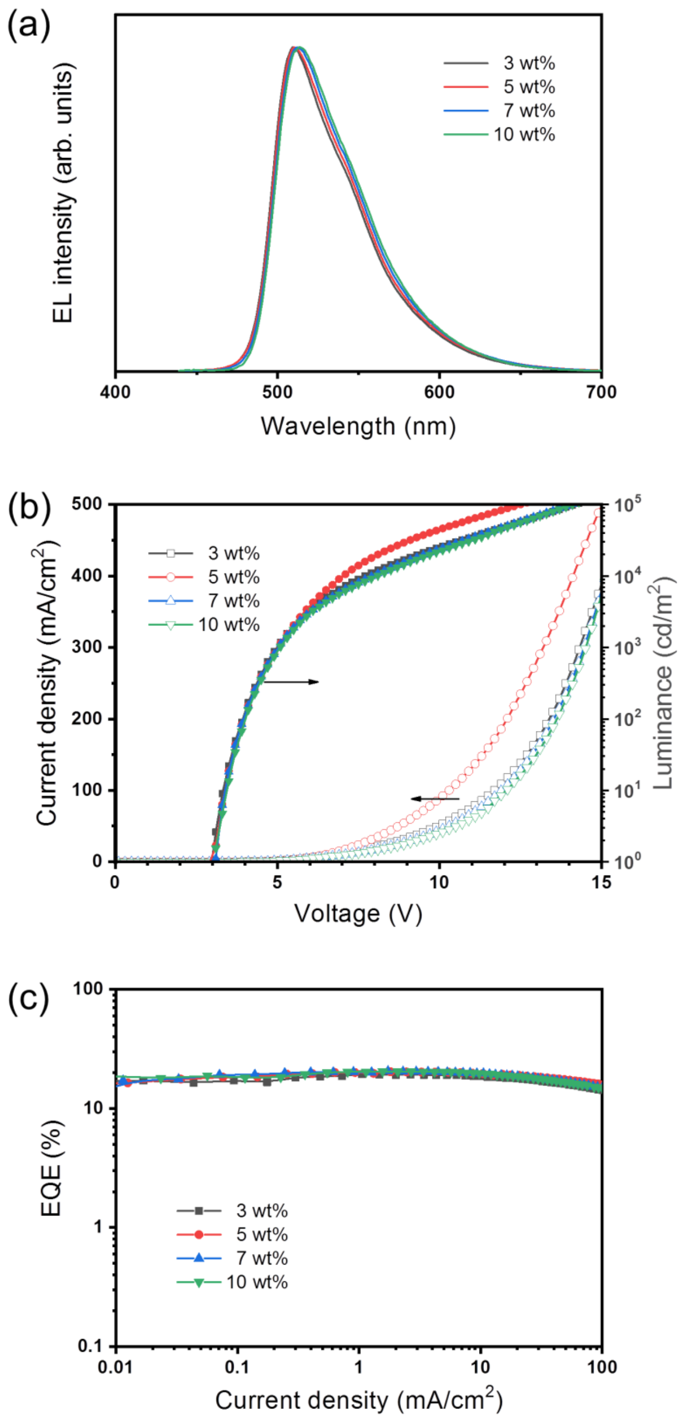

Supplementary Fig. 13 OLED characteristics. Electroluminescence spectra at 10 mA/cm^2 (**a**), current density–voltage–luminance characteristics (**b**) and EQE–current density characteristics (**c**) of the PHOLEDs with different concentrations of $Ir(ppy)_3$ in BCz-o-TRZ.

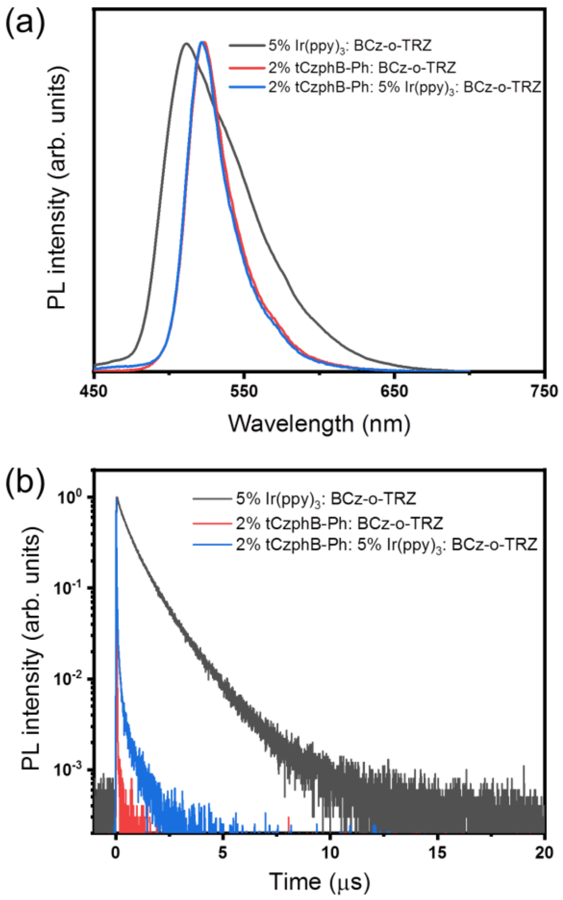

Supplementary Fig. 14 Steady-state and transient emission. Emission (**a**) and transient decay (**b**) spectra from the BCz-o-TRZ films doped with 5 wt% Ir(ppy)$_3$, 2 wt% tCzphB-Ph, and both at RT. The wavelength of the excitation light was 370 nm. The PLQY of the double-doped film (0.97) is considerably higher than that of the Ir(ppy)$_3$-single-doped film (0.67) owing to the very efficient Förster resonance energy transfer from Ir(ppy)$_3$ to tCzphB-Ph.

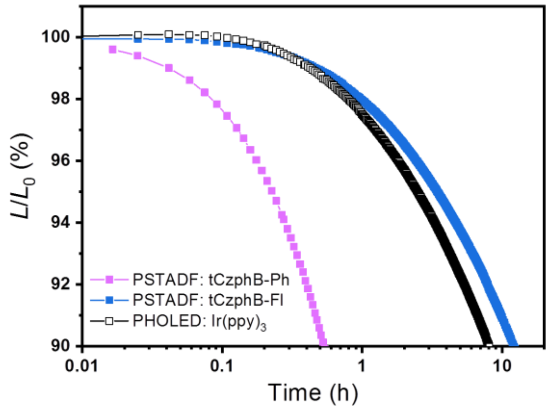

Supplementary Fig. 15 Relative luminance–operating time characteristics of the PSTADFs and PHOLED (structure I) at a fixed current density of 20 mA/cm^2. The initial luminance was 2.33×10^4, 2.45×10^4, and 1.19×10^4 cd/m^2 for the devices doping tCzphB-Ph, tCzphB-Fl, and only Ir(ppy)$_3$, respectively.

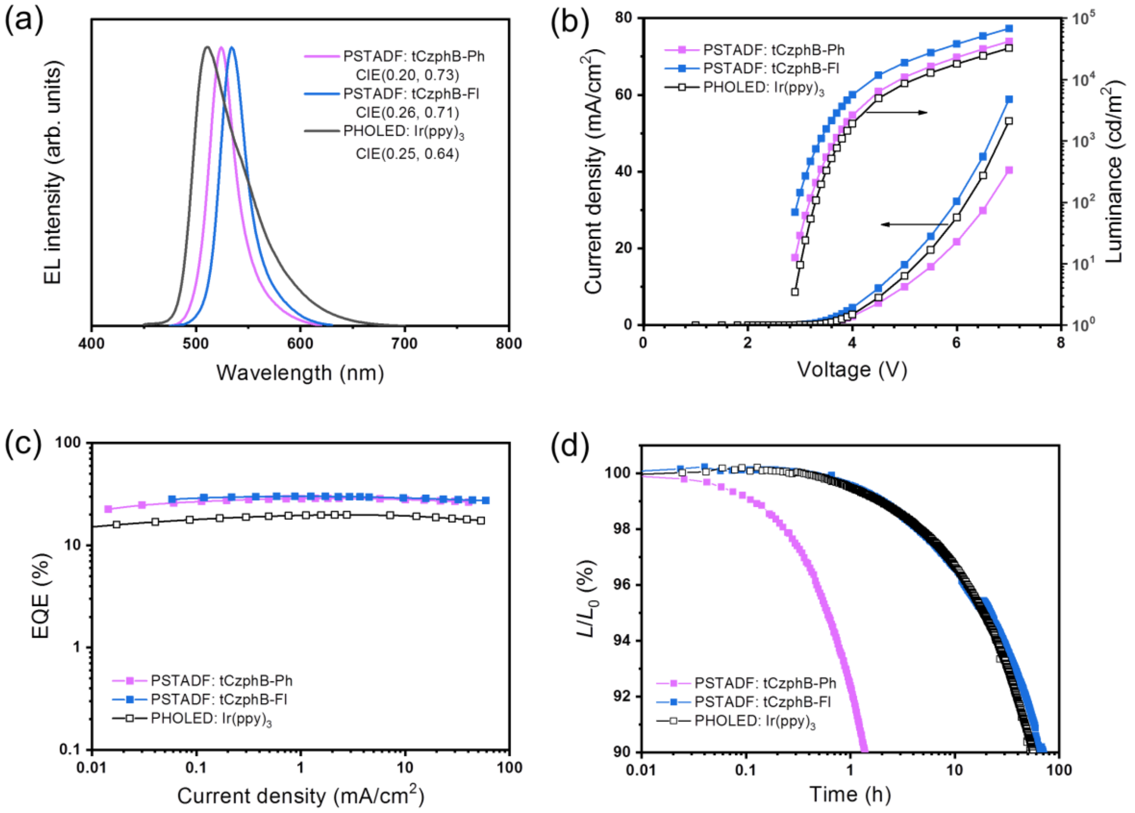

Supplementary Fig. 16 Performance of the PSTADFs and PHOLED with structure II. a EL spectra at 20 mA/cm^2. **b** Luminance–current density–voltage characteristics. **C** EQE–current density characteristics. **d** Relative luminance–operating time characteristics at a fixed current density of 20 mA/cm^2. The initial luminance was 2.14×10^4, 2.40×10^4, and 1.29×10^4 cd/m^2 for the devices doping tCzphB-Ph, tCzphB-Fl, and only Ir(ppy)$_3$, respectively.

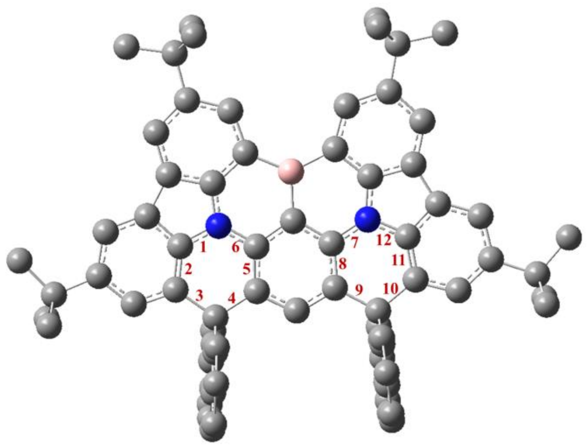

Supplementary Fig. 17 Number of the selected bonds in the tCzphBs.

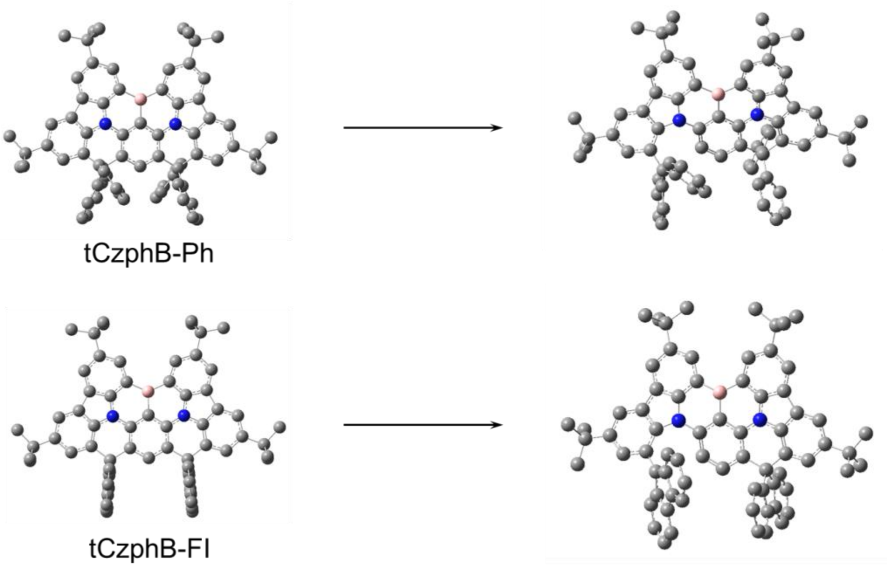

Supplementary Fig. 18 Homolytic C−C bond dissociation in tCzphBs in their ground states. The bond dissociation energies were calculated to be 4.50 eV for tCzphB-Ph and 4.63 eV for tCzphB-Fl.

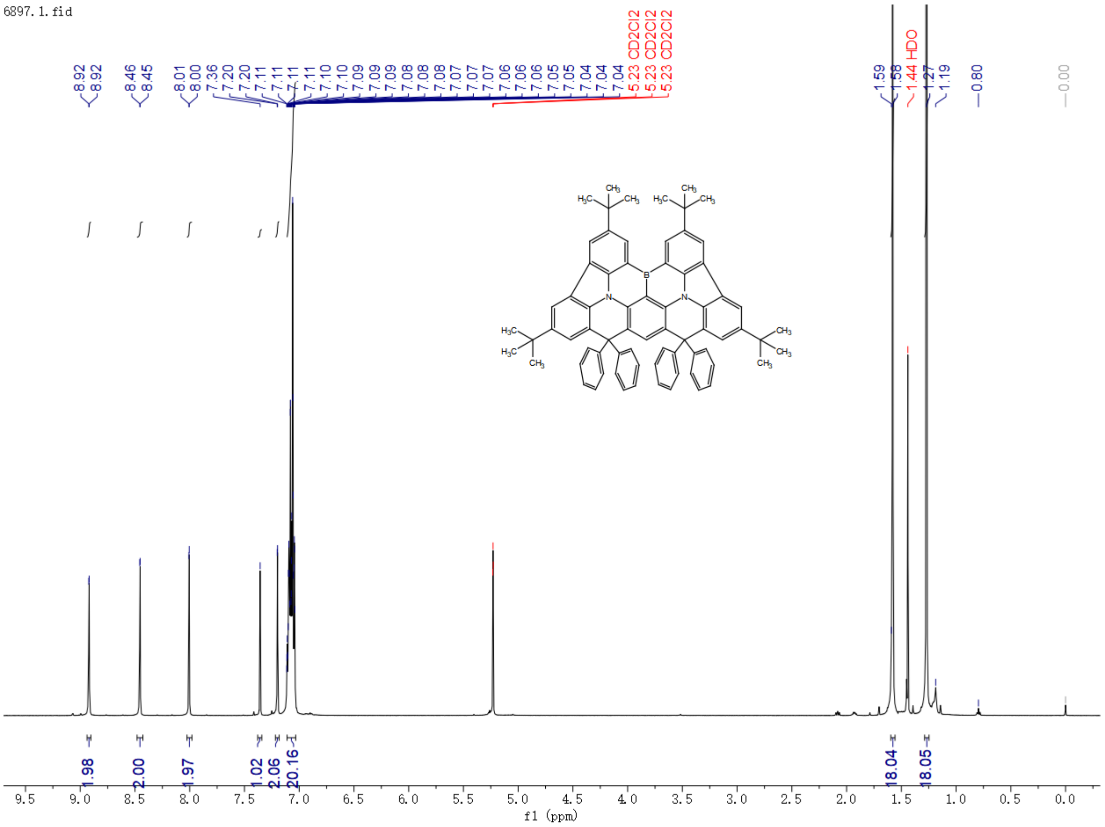

Supplementary Fig. 19 ^{1}H NMR spectrum of tCzphB-Ph in CD_2Cl_2.

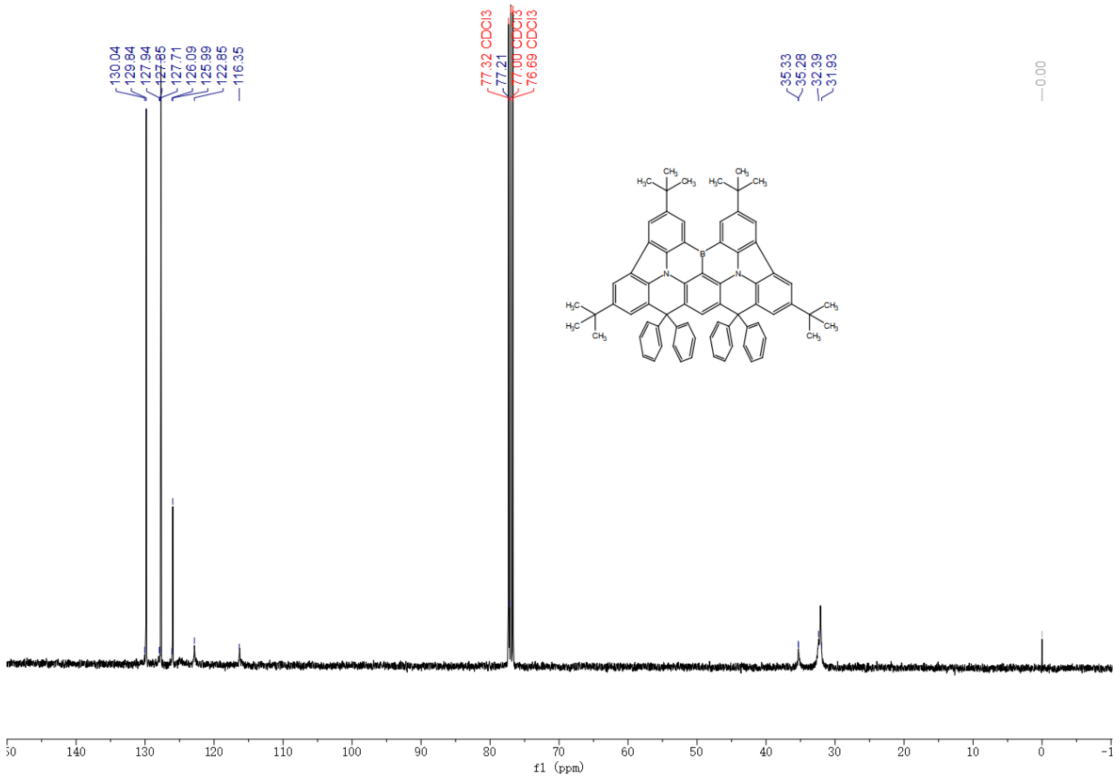

Supplementary Fig. 20 ^{13}C NMR spectrum of tCzphB-Ph in $CDCl_3$.

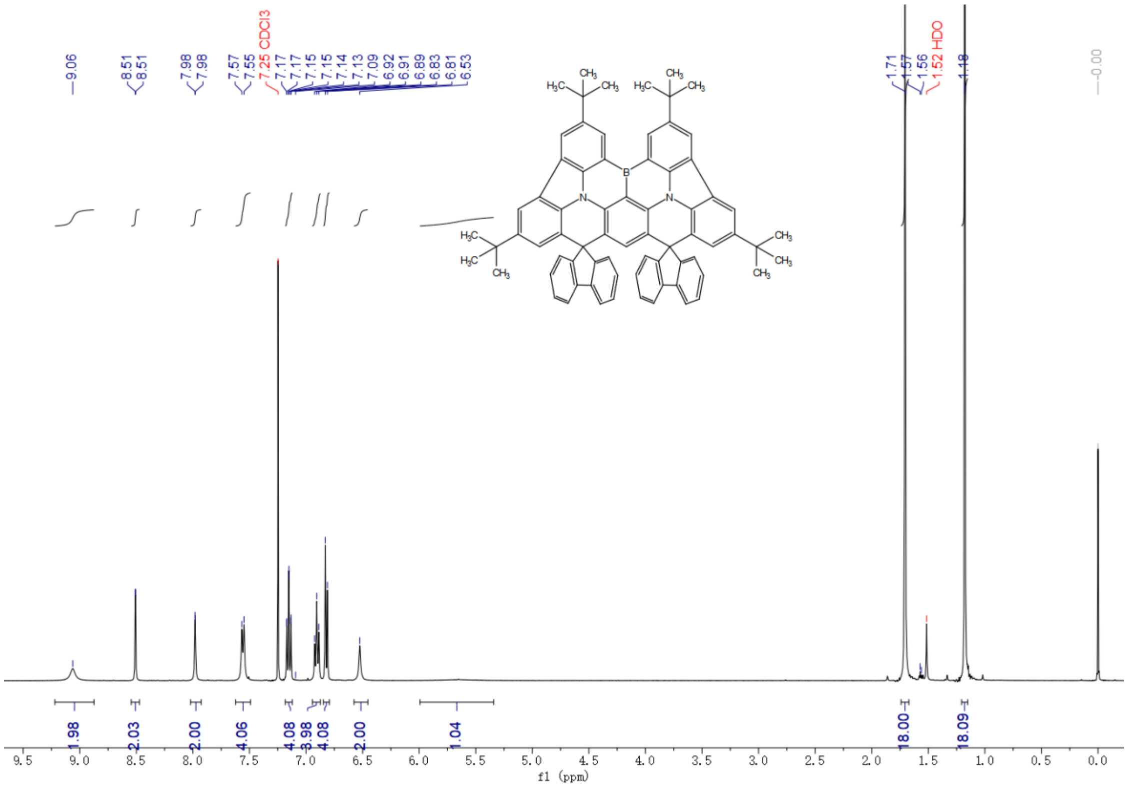

Supplementary Fig. 21 ^{1}H NMR spectrum of tCzphB-Fl in $CDCl_3$.

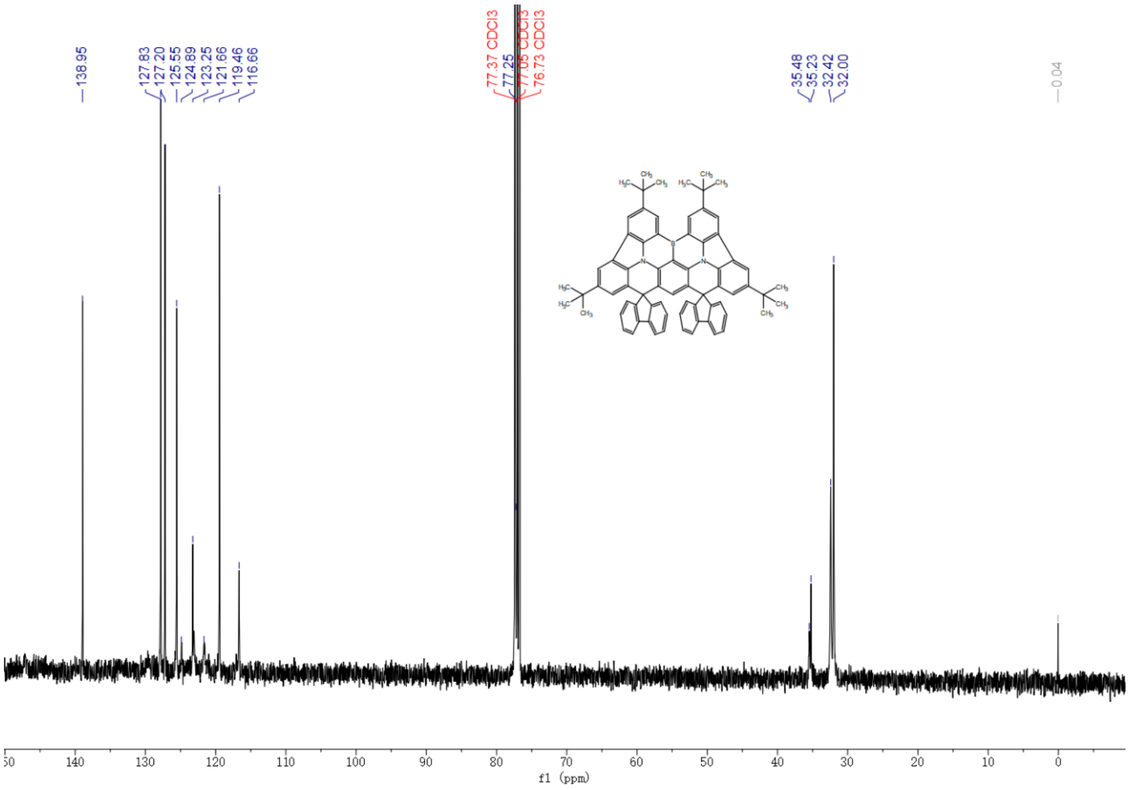

Supplementary Fig. 22 ^{13}C NMR spectrum of tCzphB-Fl in $CDCl_3$.

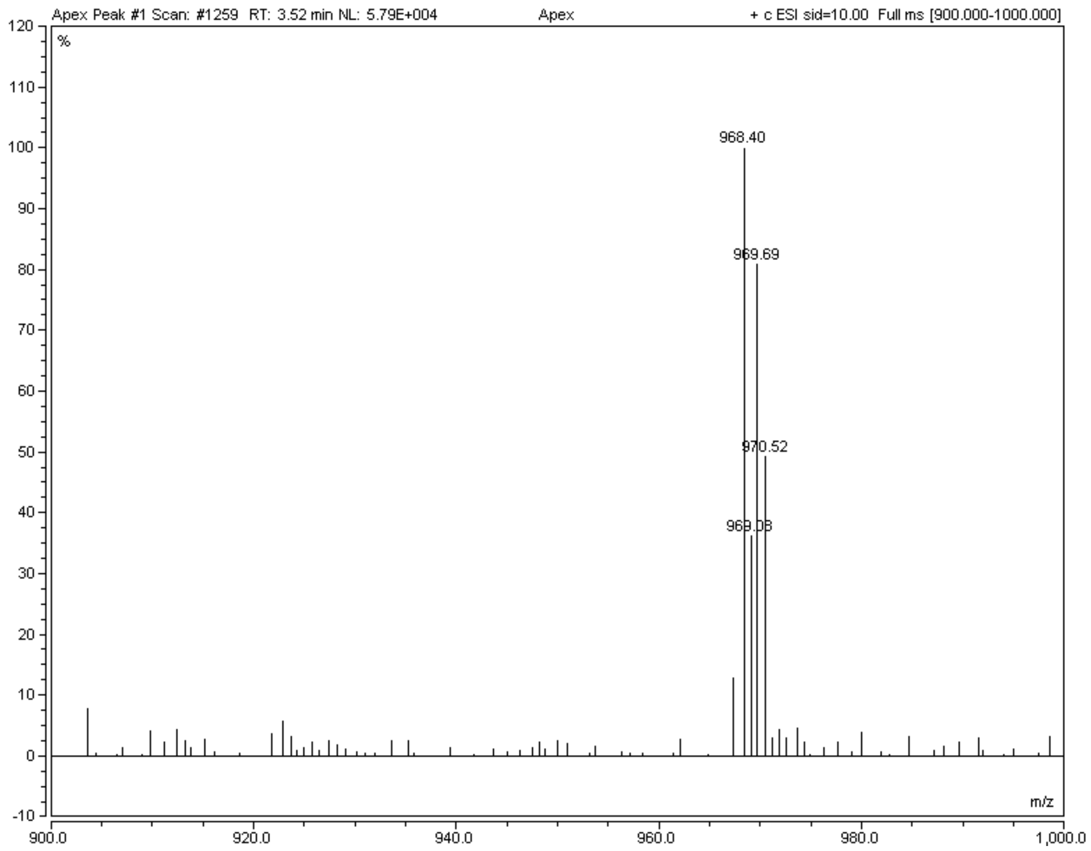

Supplementary Fig. 23 ESI-MS spectrum of tCzphB-Ph, ESI-MS (M): m/z: 968.52 $[M]^+$ (calcd: 968.40).

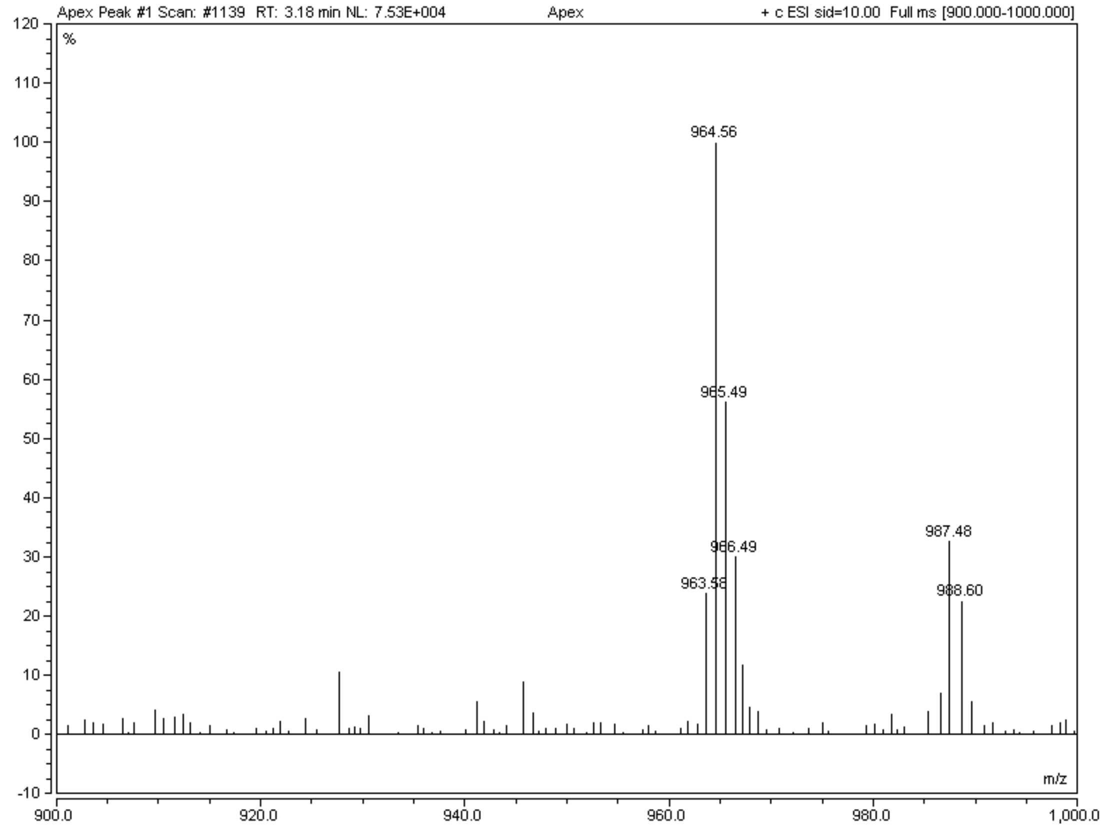

Supplementary Fig. 24 ESI-MS spectrum of tCzphB-Fl, ESI-MS (M): m/z: 964.56 $[M]^+$ (calcd: 964.49), 987.48 for $[M+Na]^+$.

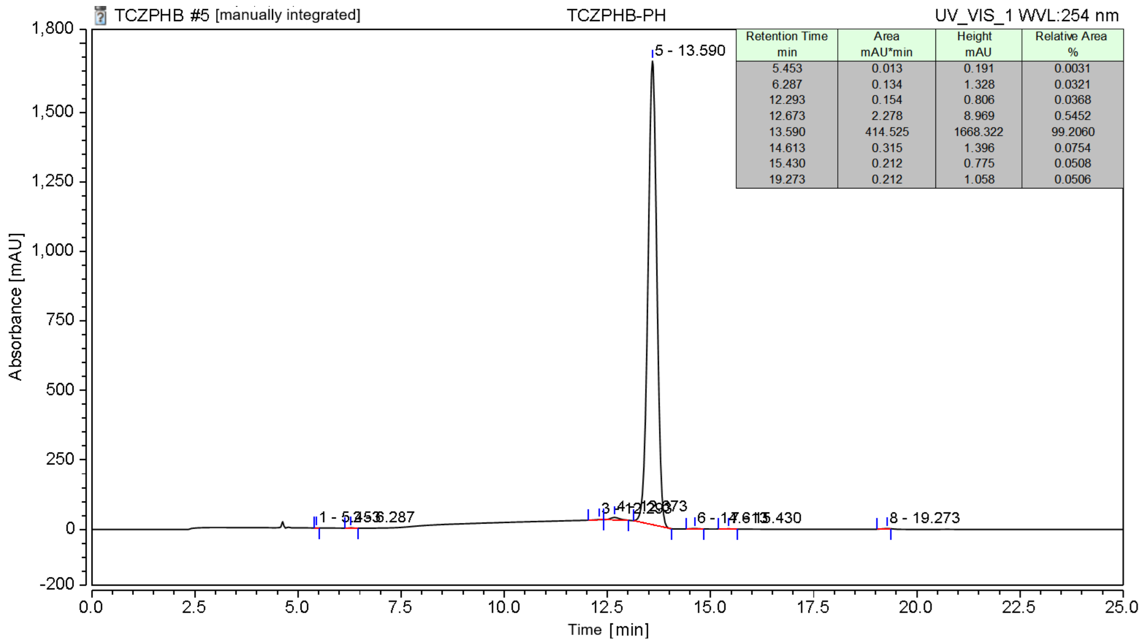

Retention Time min	Area mAU*min	Height mAU	Relative Area %
5.453	0.013	0.191	0.0031
6.287	0.134	1.328	0.0321
12.293	0.154	0.806	0.0368
12.673	2.278	8.969	0.5452
13.590	414.525	1668.322	99.2060
14.613	0.315	1.396	0.0754
15.430	0.212	0.775	0.0508
19.273	0.212	1.058	0.0506

Supplementary Fig. 25 High performance liquid chromatography (HPLC) spectrum of tCzphB-Ph (eluent: 25% THF/MeOH). The HPLC purity is 99.3%.

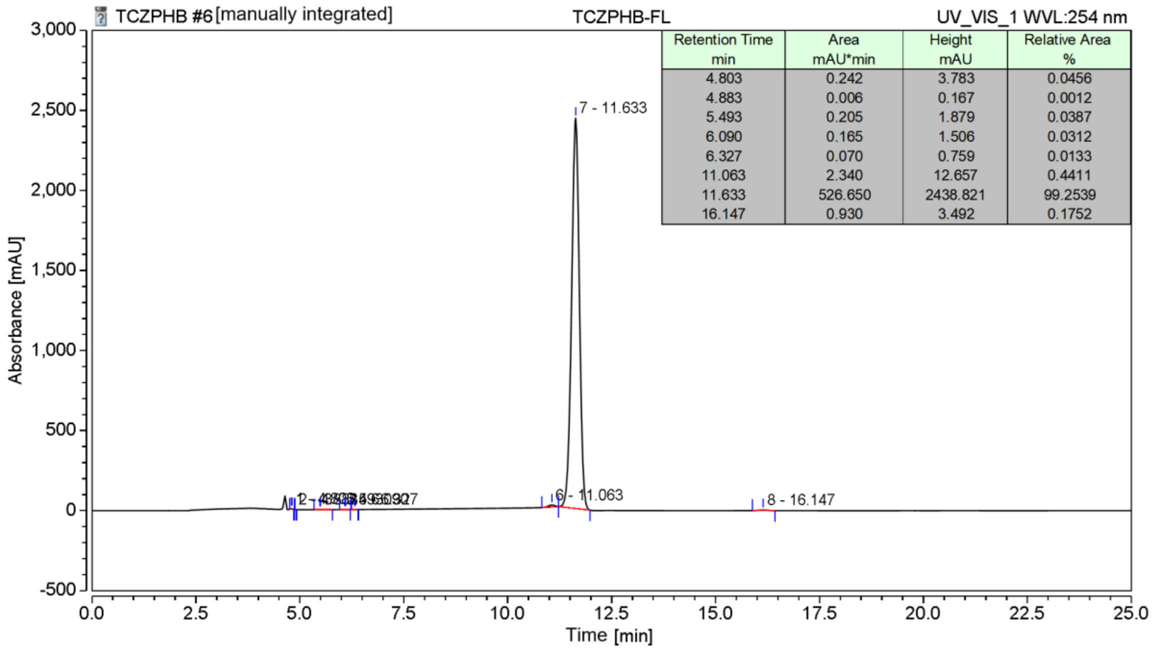

Supplementary Fig. 26 High performance liquid chromatography (HPLC) spectrum of tCzphB-Fl (eluent: 25% THF/MeOH). The HPLC purity is 99.3%

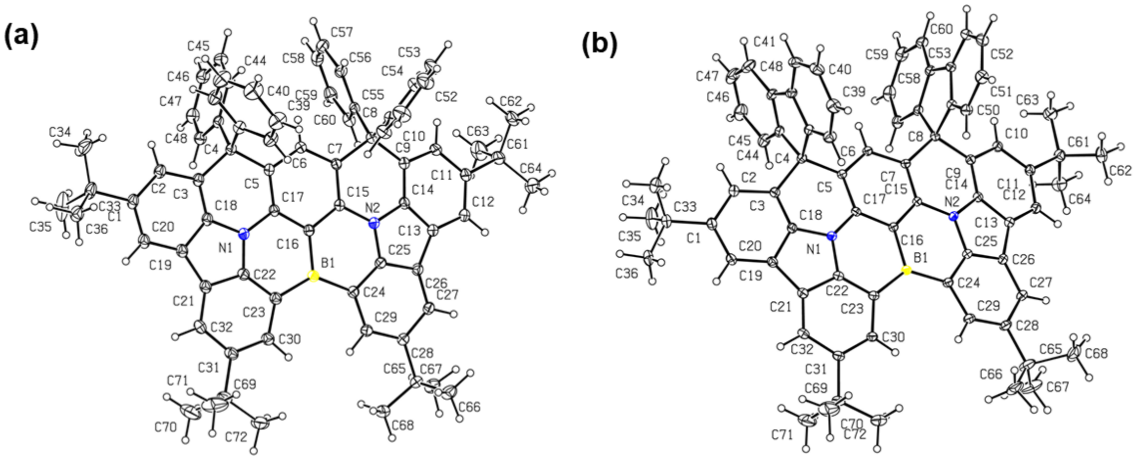

Supplementary Fig. 27 ORTEP diagram of tCzphB-Ph **(a)** and tCzphB-Fl **(b)**.

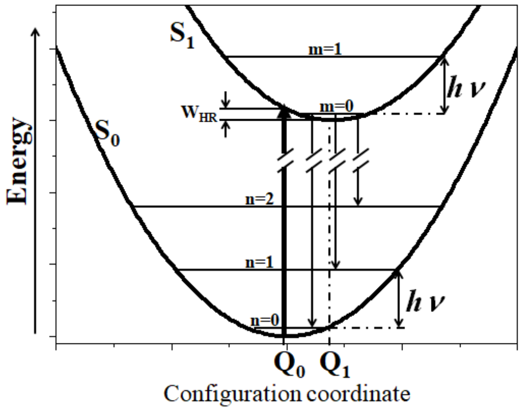

Supplementary Fig. 28 Configuration coordinate (CC) diagram of the S_1 and S_0 electronic states with vibrational states, together with the electronic transitions. The potential energy parabolae are assumed to the same harmonic oscillations with energy $h\nu$. Coordinate Q_0 and Q_1 are equilibrium coordinates of the S_1 and S_0 states, respectively. $W_{HR}=k\Delta^2/2\equiv S\hbar\omega$, where S is Huang-Rhys factor and $\Delta=Q_1-Q_0$.

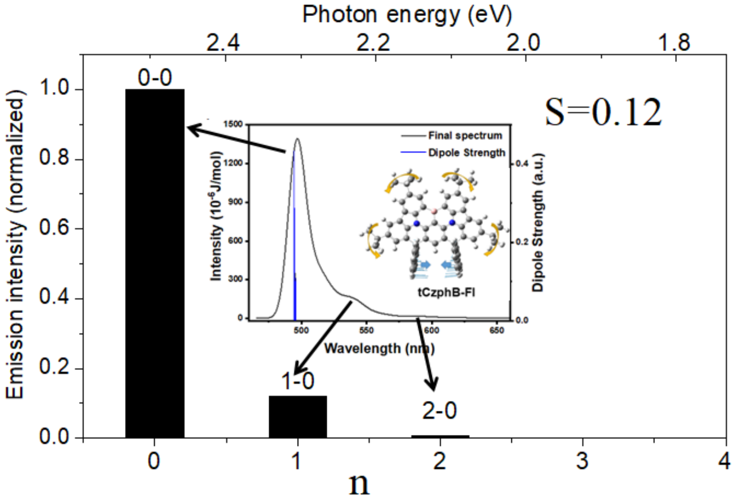

Supplementary Fig. 29 Schematic profile of the relative intensities in the 0-0, 0-1, and 0-2 vibrational transitions of tCzphB-Fl under the Huang-Rhys factor S=0.12. Inset is the PL spectrum calculated by the TD-DFT method. The 0-3 and 0-4 transitions appear at n=3 and 4, respectively. They, however, are not shown because of too small intensities compared with the 0-2 transition. The upper photon energy scale is drawn to fit to the 0-0 peak energy (2.505 eV, ~495 nm) of the calculated PL spectrum with 0-1 peak energy (2.300 eV, ~539 nm), which gives the vibrational energy $h\nu_g$ (~0.20 eV, where h is Planck's constant and ν_g is vibrational frequency) of the S_1 state. This corresponds to Δn=0.20 eV in the lower scale for integer n.

Supplementary Table 1 Summary of Franck-Condon analysis on the S_1–S_0 transition of *t*-DABNA, DtBuCzB, tCzphB-Ph, and tCzphB-Fl at the (TD) PBE1PBE/6-31G(d,p) level.

Compound	Transition ($S_1 \rightarrow S_0$)	frequency (cm^{-1})	Relative frequency (cm^{-1})	line density	dipole strength (arb. units)
t-DABNA	$0 \rightarrow 0$	23749.42	0	32.32	0.03318
	$0 \rightarrow 1^1$	23736.49	-12.9215	88.49	0.09105
	$0 \rightarrow 1^2$	23723.57	-25.843	126.7	0.1307
	$0 \rightarrow 1^3$	23710.65	-38.7645	126.3	0.1305
	$0 \rightarrow 1^4$	23697.73	-51.686	98.18	0.1017
	$0 \rightarrow 1^5$	23684.81	-64.6075	63.38	0.06581
	$0 \rightarrow 12^1 1$	23664.63	-84.791	37.73	0.03929
	$0 \rightarrow 12^1 1^2$	23651.7	-97.7125	51.78	0.05406
	$0 \rightarrow 12^1 1^3$	23638.78	-110.634	49.5	0.05177
	$0 \rightarrow 12^1 1^4$	23625.86	-123.556	36.93	0.03872
DtBuCzB	$0 \rightarrow 0$	22146.54	0	476.1	0.6463
	$0 \rightarrow 2^1$	22128.19	-18.3462	111.4	0.1518
	$0 \rightarrow 3^1$	22116.38	-30.1583	118.2	0.1613
	$0 \rightarrow 9^1$	22088.95	-57.5857	503.3	0.6907
	$0 \rightarrow 9^1 2^1$	22070.6	-75.9319	112.9	0.1554
	$0 \rightarrow 9^1 3^1$	22058.79	-87.744	127.7	0.1761
	$0 \rightarrow 9^2$	22031.37	-115.171	266.8	0.3699
	$0 \rightarrow 9^2 2^1$	22013.02	-133.518	57.25	0.07963
	$0 \rightarrow 17^1$	22001.71	-144.822	75.23	0.1049
	$0 \rightarrow 9^2 3^1$	22001.21	-145.33	69.05	0.09629
	$0 \rightarrow 9^3$	21973.78	-172.757	94.5	0.1324
	$0 \rightarrow 17^1 9^1$	21944.13	-202.408	78.91	0.1112
tCzphB-Ph	$0 \rightarrow 0$	20226.11	0	222.3	0.4338
	$0 \rightarrow 2^1$	20215.3	-10.813	147.1	0.2878
	$0 \rightarrow 2^2$	20204.49	-21.626	62.88	0.1232
	$0 \rightarrow 5^1$	20193.29	-32.8237	51.18	0.1006
	$0 \rightarrow 11^1$	20184.77	-41.341	62.59	0.1232
	$0 \rightarrow 12^1$	20177.41	-48.7005	55.46	0.1093
	$0 \rightarrow 11^1 2^1$	20173.96	-52.1541	33.18	0.06544
	$0 \rightarrow 12^1 2^1$	20166.6	-59.5135	43.19	0.08531
tCzphB-Fl	$0 \rightarrow 0$	20105.92	0	736.5	1.766
	$0 \rightarrow 6^1$	20074.22	-31.7039	317.1	0.7653
	$0 \rightarrow 6^2$	20042.51	-63.4078	68.25	0.1657
	$0 \rightarrow 15^1$	20036.96	-68.958	100.2	0.2437
	$0 \rightarrow 15^1 6^1$	20005.26	-100.6619	43.49	0.1064
	$0 \rightarrow 31^1$	19922.87	-183.054	57.06	0.1419
	$0 \rightarrow 32^1$	19910.37	-195.5531	31.22	0.07787
	$0 \rightarrow 123^1$	19419.90	-686.0244	39.27	0.1082
	$0 \rightarrow 143^1$	19299.98	-805.9399	24.27	0.06856

Supplementary Table 2 Summary of the reported green-emitting OLEDs with high color purity.

Type	Compound	λ_{EL} [nm]	$FWHM_{EL}$ [nm]	EQE_{max} [%]	L_{max} [kcd/m^2]	CIE_{EL} [x, y]	Ref.
Fluorescence	C-545T	500	60	N.A.	12	[0.31, 0.65]	[1]
	tPhBODIPY	520	32	19.0	N.A.	[0.26, 0.67]	[2]
Phosphorescence	PtN7N	518	24	22	10	[0.27, 0.67]	[3]
TADF	4CzIPN	507	80	22.3	20	[0.29, 0.58]	[4]
	CzDBA	528	75	37.8	<100	[0.31, 0.61]	[5]
MR-TADF	2F-BN	501	40	22	N.A.	[0.16, 0.60]	[6]
	DtBuPhCzB	508	33	25.5	38	[0.20, 0.65]	[7]
	m-Cz-BNCz	520	44	27.0	26	[0.23, 0.69]	[8]
	BBCz-G	519	50	31.8	<20	[0.23, 0.65]	[9]
	AZA-BN	527	30	28.2	10	[0.27, 0.69]	[10]
This work MR-TADF	tCzphB-Fl	535	26	26.2	37	[0.26, 0.72]	
	tCzphB-Ph	527	24	29.3	32	[0.21, 0.75]	
	tCzphB-Ph	526	27	31.3	520	[0.21, 0.74]	

Supplementary Table 3 Length and bond order of the selected bonds (Supplementary Fig. 16) in the tCzphBs in their ground and excited states.

		Bond length (Å)				Laplacian bond order			
Bond number		**3**	**4**	**9**	**10**	**3**	**4**	**9**	**10**
tCzphB-Ph	S_0	1.54414	1.55634	1.55634	1.54414	0.896	0.853	0.853	0.896
	S_1	1.53835	1.54905	1.54905	1.53835	0.914	0.876	0.876	0.914
tCzphB-Fl	S_0	1.53663	1.54623	1.54623	1.53663	0.906	0.874	0.874	0.906
	S_1	1.52923	1.53839	1.53839	1.52923	0.930	0.898	0.898	0.930

Note: The lengths of bond **4** and **9** in tCzphB-Ph are larger than the normal length of a C−C δ-bond (~1.54 Å).[11]

Supplementary Table 4 Crystal data and structure refinement for tCzPhB-Ph.

Empirical formula	$C_{72}H_{65}BN_2$
Formula weight	969.07
Temperature/K	170.0
Crystal system	triclinic
Space group	P-1
a/Å	15.1926(6)
b/Å	15.9860(7)
c/Å	17.0622(7)
α/ °	64.4760(10)
β/ °	69.0720(10)
γ/ °	66.3730(10)
Volume/Å^3	3339.6(2)
Z	2
ρ_{calc}g/cm^3	0.964
μ/mm^{-1}	0.055
F(000)	1032.0
Crystal size/mm^3	0.42 × 0.28 × 0.2
Radiation	MoKα (λ = 0.71073)
2Θ range for data collection/°	4.474 to 61.844
Index ranges	$-21 \le h \le 19$, $-23 \le k \le 23$, $-24 \le l \le 24$
Reflections collected	82715
Independent reflections	20672 [R_{int} = 0.0394, R_{sigma} = 0.0401]
Data/restraints/parameters	20672/357/781
Goodness-of-fit on F^2	1.037
Final R indexes [I>=2σ (I)]	R_1 = 0.0606, wR_2 = 0.1601
Final R indexes [all data]	R_1 = 0.0873, wR_2 = 0.1750
Largest diff. peak/hole / e Å^{-3}	0.44/-0.34

Supplementary Table 5 Crystal data and structure refinement for tCzPhB-Fl.

Empirical formula	$C_{72}H_{61}BN_2$
Formula weight	965.03
Temperature/K	170.0
Crystal system	monoclinic
Space group	$P2_1/c$
a/Å	16.2161(6)
b/Å	31.9970(11)
c/Å	12.2815(3)
α/ °	90
β/ °	104.3510(10)
γ/ °	90
Volume/Å^3	6173.6(3)
Z	4
ρ_{calc}g/cm^3	1.038
μ/mm^{-1}	0.059
F(000)	2048.0
Crystal size/mm^3	0.32 × 0.23 × 0.2
Radiation	MoKα (λ = 0.71073)
2Θ range for data collection/°	4.266 to 66.2
Index ranges	$-24 \le h \le 24, -49 \le k \le 44, -15 \le l \le 18$
Reflections collected	119473
Independent reflections	20615 [R_{int} = 0.0501, R_{sigma} = 0.0420]
Data/restraints/parameters	20615/204/775
Goodness-of-fit on F^2	1.030
Final R indexes [I>=2σ (I)]	R_1 = 0.0616, wR_2 = 0.1725
Final R indexes [all data]	R_1 = 0.1005, wR_2 = 0.2054
Largest diff. peak/hole / e Å^{-3}	0.50/-0.81

Supplementary Note 1. |Appearance of Narrow Emission Bands.

Three peaks are found in the simulated PL spectra of tCzphB-Ph, tCzphB-Fl, and DtBuCzB. For example, tCzphB-FI shows three simulated PL peaks at 500 nm (20000 cm^{-1}), 540 nm (18500 cm^{-1}), and 590 nm (16900 cm^{-1}), with almost the same separation of about 1550 cm^{-1} between the two neighboring components, where the lowest-energy component intensity is considerably small (Fig. 3d). The same was found in tCzphB-Ph (or DtBuCzB) that shows peaks at 20110 (or 22000), 18580 (20450), and 16900 (18900) cm^{-1}, with separation of about 1600 (or 1550) cm^{-1} (Fig. 3c and Supplementary Fig. 7b). These spectra are not inconsistent with the observed PL spectra (Fig. 2). When we use a model of harmonic parabola potentials for the excited and ground states with the same force constants (Supplementary Fig. 28) and vibrational modes, the three peaks are attributable to the 0-0, 0-1, and 0-2 vibrational transition components (Supplementary Fig. 29). Since the intensity ratio of the 0-1 component to the 0-0 one ($I_{0\text{-}1}/I_{0\text{-}0}$) is given by the Huang-Rhys factor S, the S factors are obtained to be 0.12, 0.13, and 0.11 for tCzphB-FI, tCzphB-Ph, and DtBuCzB, respectively (Fig. 3 and Supplementary Fig. 7).

This model predicts that the 0-3 vibrational component intensity is 0.000288 times of the 0-0 component band for tCzphB-FI with S=0.12. This intensity is too weak to be found in the actual spectrum measurement as the 0-2 component (Fig. 2). Unlike tCzphB-Ph, Alq_3 exhibits large Huang-Rhys factor of S~2.6, from which Alq_3 never shows a clear vibrational structure at RT. If such a large S factor would be obtained for tCzphB-Ph, this molecule would have never shown the narrow EL band. In this way we understand that small Huang-Rhys factor is responsible to the PL/EL band narrowing.

The Huang-Rhys factor S is given by $S\hbar\omega = k\Delta^2/2$, with force constant k ($=M\omega^2$), where M is effective mass of molecule. A small S value is obtained by small potential shift Δ ($= Q_1 - Q_0$) of the equilibrium coordinate Q_1 of the S_1 state from the equilibrium coordinate Q_0 of the S_0 state.[12] Supplementary Fig. 28 is drawn using the Franck-Condon (FC) factor F_{n0} that is related with the coupling between the different vibrational states. In the present case of the transition from the vibrational 0 state of the excited state to the n (=0, 1, 2, 3…) vibrational states of the ground state, the FC factor is given by

$F_{n0}=e^{-S}S^n/n!$.[11] The small Q_1-Q_0 separation Δ due to small electron-phonon coupling is better to obtain narrow band spectrum by the following two reasons. The first is that the whole spectrum is predominantly determined by the 0-0 component and the contribution of the neighboring 0-1 component is nearly negligible. The relation of $I_{0\text{-}1}/I_{0\text{-}0}=S$ indicates that a small 0-1 component intensity lead to a small S. The second is that the 0-1 component is located far from the 0-0 component which is located at $h\nu$ (or $\hbar\omega$) from the 0-0 component (Supplementary Fig. 28), since $\hbar\omega$ increases with decrease of S from the relation of $S\hbar\omega=k\Delta^2/2$, *i.e.*, $\hbar\omega=k\Delta^2/2S$. In summary, the contribution of the neighboring 0-1 component to the whole band width can be negligible because of the small Huang-Rhys factor S of 0.13.

Such a small S value is possible for the multi-resonance structured molecules like tCzphB-Ph with rigid chemical structure. Small S is generated by small Δ, *i.e.*, small shift of the equilibrium position of the S_1 state from that of the ground state. One of the reasons for small shift is the rigidness which does not allow a large lattice relaxation after excitation into the excited state. The detailed analysis with evidence is under consideration.

It is noted that the simulated PL spectrum of tCzphB-Ph (Fig. 3d) consists of a lot of vibrational components (more than 850 components) due to various vibrational modes. For example, eight components are contained in a limited 60 cm^{-1} range of 20226.11−20166.6 cm^{-1} (Supplementary Table 1), which are caused by the modes-2, 5, 11, and 12 due to the rocking, twisting (rotation of *t*Bu-group), twisting (rotation of spiro-carbons with two phenyl groups), and scissoring (for whole molecule) vibrations, respectively, with different dipole strengths. These modes do not induce a vibration due to movements of atoms in boranaphtho[3,2,1-de]anthracene with one boron and two nitrogen atoms, suggesting a contribution to suppress the electron-phonon coupling in the $S_1 \rightarrow S_0$ transition.

Supplementary Note 2. | Emission Band Narrowing in the cHex Solvent

Molecule tCzphB-Ph shows a PL spectrum with FHWM of 21 nm when it is dissolved in toluene (Fig. 2b), while a wider FWHM by 9 nm is observed in the CBP film doped with 2 wt% tCzphB-Ph (Fig. 2c). The PL band of tCzphB-Ph molecule exhibits a red shift when solvent is changed from cHex to toluene to THF to DCM, together with broadening of FWHM (Fig. 2c), *e.g.*, FWHM=14 nm in cHex solvent. Considerably narrow FWHM is obtained from tCzphB-Ph. The solvatochromism of the emission band peak shift, which follows the *Lippert-Mataga equation*, is based on the dipole-dipole coupling between the dipole moment of the emitter molecule and the dipole moment of the host molecule. A wider PL band with FWHM of 21 nm is observed in toluene compared with FWHM of 14 nm in cHex. This is due to the μ_G=0.31 D and ε_r=2.38 for toluene, which are larger than the $\mu_G \approx 0$ D and ε_r=2.02 for cHex, where μ_G and ε_r are the dipole moment in the ground state and relative dielectric constant, respectively.

Larger dipole-dipole interaction between the tCzphB-Ph molecule and toluene solvent induces larger Δ shift because of reorientation of dipole moment of toluene molecules surrounding tCzphB-Ph molecule, compared with the non-polar cHex molecule. As a result, toluene gives rise to a larger S value than cHex. Consequently, unlike in cHex, the contribution of the 0-1 component to the broadening of the whole emission spectrum is negligible in toluene solvent. The closer location of the 0-1 component to the 0-0 component also contributes to the broadening since the distance $\hbar\omega$ between the two components becomes small and leads to spectral overlap, which is caused by smaller S from the above-mentioned relation $\hbar\omega = k\Delta^2/2S$. In this way the small FWHM of 14 nm observed in cHex than FWHM of 21 nm in toluene is understood.

Supplementary Note 3. |Appearance of Long TADF Lifetime

According to the Fermi's golden rule, the reversed intersystem crossing rate between the initial (T_1) and final (S_1) states is expressed by

$$k_{RISC}=(2\pi/\hbar)\langle\varphi_{S1}(r)\chi_{S1,n}(Q)|H_{SO}(r)H_{vib}(Q)|\varphi_{T1}(r)\,\chi_{T1,n}(Q)\rangle^2\rho(E_{S1})$$

$$k_{RISC}=(2\pi/\hbar)\langle\varphi_{S1}(r)|H_{SO}(r)|\varphi_{T1}(r)\rangle^2\rho_{FCWD}$$

where $H_{SO}(r)$ and $H_{vib}(Q)$ are the spin-orbit and electron-phonon interaction Hamiltonians, respectively, while $\varphi_{S1}(r)$ and $\chi_{S1,n}(Q)$ are the electronic wavefunction of the S_1 state and the vibrational wavefunction of the *n*-th vibrational state in S_1, respectively. $\rho(E_{S1})$ is the density of the final S_1 state, and ρ_{FCWD} is the Frank-Condon-weighted density of states that is derived from the Franck-Condon factor $\langle\chi_{S1,n}(Q)|\,\chi_{T1,n}(Q)\rangle^2$. The Marcus-Levich-Jortner Theory based on the Franck-Condon Principle provides the following formula for ρ_{FCWD}:[13]

$$\rho_{FCWD} = \frac{1}{\sqrt{4\pi\lambda_M k_B T}}\sum_{n=0}^{\infty}\exp(-|S_{eff})\frac{S_{eff}^n}{n!}\exp\left(-\frac{(\Delta E_{ST}+n\hbar\omega_{eff}+\lambda_M)^2}{4\lambda_M k_B T}\right)$$

where S_{eff} is the effective Huang-Rhys factor of the effective mode with effective frequency ω_{eff}, which is responsible to the strength of the electron-phonon coupling, and λ_M is the reorganization energy, *i.e.*, the energy required to bring the system in the S_1 to the minimum structure of the T_1 state. The theory takes into account the overlap of the vibrational state between the S_1 state with vibrational state $n=n'$ and the T_1 state with $n=n''$ where the two states have the same energy, allowing the electronic reverse intersystem crossing transition from the T_1 state to the S_1 state. The part $\exp(S_{eff})n!/S^n_{eff}$ (defined as $C(S_{eff})$) involved the Huang-Rhys factor S_{eff} gives a large value if S_{eff} is small. Both tCzphB-Ph and tCzphB-FI give $S_{eff} \approx 0.12$ (Fig. 2d and Supplementary Fig. 7), and then gives the intensity for the 0-0 component (n=0), $C(S_{eff}=0.12) \approx 0.135$. Classical charge transfer (CT) molecules have a large S_{eff} and a large $C(S_{eff})$ as a result, *e.g.*, $S_{eff}=2.0$ and $C(S_{eff}=2) \approx 14.8$.

Becasue k_{RISC} is proportional to k_{TADF}, the delayed fluorescence lifetime τ_{TADF} is proportional to $\exp(-S_{eff})S^n_{eff}/n!$. Consequently, the two tCzphB-Ph and tCzphB-FI compounds are suggested to have much longer τ_{TADF}, about by 109.6 (=14.8/0.135) times longer than the classical CT-type molecules. In this way, we understand the reason why

the present compounds exhibit long τ_{TADF} from the component related with the Huang-Rhys factor in the equation for ρ_{FCWD} constructed by the Marcus–Levich–Jortner theory.

Supplementary Note 4. |Synthesis of MR-TADF Emitters.

Synthesis of 9,9'-(4,6-dibromo-1,3-phenylene)bis(3,6-di-tert-butyl-9H-carbazole) (**A**):

60 mL anhydrous DMF was added into a 200 ml Schlenk flask, which contained 3,6-di-*tert*-butyl-9H-carbazole (11.3 g, 40.5 mmol), 1,5-dibromo-2,4-difluorobenzene (5.0 g, 18.4 mmol) and Caesium carbonate (18.0 g, 55.2 mmol), under a nitrogen atmosphere. The solution was refluxed with magnetic stirring for 24 h and then cooled down. The resulted solution was slowly poured into ice water (1 L) with stirring with glass rod. The powder solid was filtered out and dried in vacuum, and then further purified by column chromatography with a mixture eluent of ethyl acetate/petroleum ether (1:10), resulting in a white solid (**A**) of 13.2 g. Yield: 91 %. ^{1}H NMR (400 MHz, chloroform-d) δ 8.36 (s, 1H), 8.11 (d, *J* = 1.8 Hz, 4H), 7.54 (s, 1H), 7.46 (dd, *J* = 8.6, 1.9 Hz, 4H), 7.08 (d, *J* = 8.6 Hz, 4H), 1.44 (s, 36H).

Synthesis of 3,6,12,15-tetra-*tert*-butyl-8,8,10,10-tetraphenyl-8,10-dihydroindolo [1,2,3-*fg*]indolo [3',2',1':8,1]quinolino[3,2-*b*]acridine (B1):

A solution of *n*-butyllithium in *n*-hexane (4.7 mL, 2.5 M, 11.7 mmol) was slowly added dropwise into a solution of compound **A** (4.0 g, 5.1 mmol) in anhydrous tetrahydrofuran (THF) (100 mL) at -78 ℃ under a nitrogen atmosphere. After stirring at -78 ℃ for 2 h, a solution of benzophenone (2.3 g, 12.7 mmol) in anhydrous THF was slowly added under a nitrogen atmosphere. Then the reaction mixture was slowly warmed to RT and stirred overnight. 20 mL diluted HCl solution (1 M), 400 mL distilled water and 150 mL ethyl acetate was added into the reaction mixture. The aqueous layer was separated and extracted with ethyl acetate three time (100 mL). The combined organic layers were dried with sodium sulphate, filtered. After removal of solvent under reduced pressure, the crude product was dissolved in anhydrous DCM (100 mL), and then 47% Boron trifluoride-diethyl etherate (2 mL) was added slowly at RT. The reaction mixture was stirred overnight, and slowly quenched by 100 mL $NaHCO_3$ aqueous solution, and then aqueous layer was separated and extracted with DCM (50 mL). The combined organic layers were dried with sodium sulphate, filtered, and concentrated by rotary evaporation. The product was further purified by column chromatography with using a

mixture eluent of DCM/petroleum ether (1: 20), afforded **B1** as a white solid (3.0 g, 61%). ^{1}H NMR (400 MHz, chloroform-d) δ 8.67 (s, 1H), 8.24–8.04 (m, 4H), 7.93 (d, *J* = 1.7 Hz, 2H), 7.70 (dd, *J* = 8.7, 2.0 Hz, 2H), 7.15–7.06 (m, 14H), 7.01–6.87 (m, 8H), 6.75 (s, 1H), 1.51 (s, 18H), 1.34 (s, 18H).

Synthesis of 3',6',12',15'-tetra-*tert*-butyldispiro[fluorene-9,8'-indolo[1,2,3-*fg*]indolo [3',2',1':8,1]quinolino[3,2-*b*]acridine-10',9''-fluorene] (B2)

A similar procedure like synthesizing **B1** was carried out except replacing benzophenone with flourenone (2.3 g, 12.7 mmol), resulting in a white solid (3.2 g, 65 %). ^{1}H NMR (400 MHz, chloroform-d) δ 8.78 (s, 1H), 8.35 (d, *J* = 8.7 Hz, 2H), 8.20 (d, *J* = 2.0 Hz, 2H), 7.92–7.78 (m, 4H), 7.55 (d, *J* = 7.6 Hz, 4H), 7.20 (td, *J* = 7.3, 1.3 Hz, 4H), 6.99–6.83 (m, 8H), 6.50 (d, *J* = 1.6 Hz, 2H), 5.65 (s, 1H), 1.57 (s, 18H), 1.15 (s, 18H).

Synthesis of 19-bromo-3,6,12,15-tetra-*tert*-butyl-8,8,10,10-tetraphenyl-8,10-dihydroindolo[1,2,3-*fg*]indolo[3',2',1':8,1]quinolino[3,2-*b*]acridine (C1):

After the chloroform solution (50 mL) of **B1** (2.0 g, 2.1 mmol) was cooled to 0 ℃ with strict exclusion of light, *N*-bromosuccinimide (NBS) (0.44 g, 2.5 mmol) was added stepwise into it. The reaction was maintained at 0 ℃ for 30 min and the stirring continued overnight at RT. The reaction mixture was poured into water (300 mL) and extracted with DCM, and the combined organic layers were dried with sodium sulphate, filtered, and concentrated by rotary evaporation. The product was further purified by column chromatography with using a mixture eluent of DCM/petroleum ether (1: 20), afforded **C1** as a yellowish solid (2.1 g, 97%). The obtained product was directly subjected to the next step without further purification.

Synthesis of 19'-bromo-3',6',12',15'-tetra-*tert*-butyldispiro[fluorene-9,8'-indolo [1,2,3-*fg*]indolo[3',2',1':8,1]quinolino[3,2-*b*]acridine-10',9''-fluorene] (C2):

A similar procedure like synthesizing **C1** was carried out except replacing **B1** with **B2** (2.0 g, 2.1 mmol), resulting in a yellowish solid (2.1 g, 95%). The obtained product was directly subjected to the next step without further purification.

Synthesis of tCzphB-Ph:

A solution of *n*-butyllithium in *n*-hexane (1.1 mL, 2.5 M, 2.8 mmol) was slowly added dropwise into a solution of compound **C1** (2.0 g, 1.9 mmol) in anhydrous tert-butylbenzene (80 mL) at -78 ℃ under a nitrogen atmosphere. When the addition was finished, the reaction mixture was slowly warmed to -40 ℃ and stirred at -40 ℃ for 2 h. After boron tribromide (0.5 mL, 5.6 mmol) has been added at -78 ℃, the reaction mixture was slowly warmed to RT and stirred overnight. *N*,*N*-diisopropylethylamine (0.8 mL, 5.6 mmol) was added at 0 ℃ and then the reaction mixture was stirring at 130 ℃ for 4 h. The reaction mixture was cooled to RT and quenched with 2 mL methanol. The reaction mixture was poured into water (300 mL) and extracted with DCM, and the combined organic layers were dried with sodium sulphate, filtered, and concentrated by rotary evaporation. The product was further purified by column chromatography with using a mixture eluent of DCM/petroleum ether (1:20), then recrystallized using DCM/methanol, afforded **tCzphB-Ph** as a yellow solid. (1.1 g, 61%). ESI-MS (M): m/z: 968.40 $[M]^+$ (calcd: 968.52). Anal. Calcd for $C_{72}H_{65}BN_2$: C, 89.23; H, 6.76; B, 1.12; N, 2.89. Found: C, 89.36; H, 6.77; N, 2.48. (B is not analyzed by elemental analysis). ^{1}H NMR (500 MHz, methylene chloride-d_2) δ 8.92 (d, J = 1.7 Hz, 2H), 8.45 (d, J = 1.6 Hz, 2H), 8.01 (d, J = 1.4 Hz, 2H), 7.36 (s, 1H), 7.20 (d, J = 1.4 Hz, 2H), 7.11 – 7.03 (m, 20H), 1.58 (s, 18H), 1.27 (s, 18H). ^{13}C NMR (101 MHz, chloroform-d) δ 130.04, 129.84, 127.94, 127.85, 127.71, 126.09, 125.99, 122.85, 116.35, 77.21, 35.33, 35.28, 32.39, 31.93.

Synthesis of tCzphB-Fl:

A similar procedure like synthesizing **tCzphB-Ph** was carried out except replacing **C1** with **C2** (2.0 g, 1.9 mmol), resulting in a yellow solid (0.9 g, 55 %). ESI-MS (M): m/z: 964.56 $[M]^+$ (calcd: 964.49). Anal. Calcd for $C_{72}H_{61}BN_2$: C, 89.61; H, 6.37; B, 1.12; N, 2.90. Found: C, 89.67; H, 6.47; N, 2.67. (B is not analyzed by elemental analysis). ^{1}H NMR (400 MHz, chloroform-d) δ 9.06 (s, 2H), 8.51 (d, J = 1.5 Hz, 2H), 8.05–7.84 (m, 2H), 7.56 (d, J = 7.7 Hz, 4H), 7.15 (td, J = 7.5, 1.2 Hz, 4H), 6.91 (t, J = 7.4 Hz, 4H), 6.82 (d, J = 7.5 Hz, 4H), 6.53 (s, 2H), 5.65 (s, 1H), 1.71 (s, 18H), 1.18 (s, 18H). ^{13}C NMR (101 MHz, chloroform-d) δ 138.95, 127.83, 127.20, 125.55, 124.89, 123.25, 119.46, 116.66,

77.25, 35.48, 35.23, 32.42, 32.00.

Supplementary References

1. Lee, M.-T. *et al.* Efficient green coumarin dopants for organic light-emitting devices. *Org. Lett.* **6**, 1241–1244 (2004).
2. Song, X., Zhang, D., Zhang, Y., Lu, Y. & Duan, L. Strategically modulating carriers and excitons for efficient and stable ultrapure-green fluorescent OLEDs with a sterically hindered BODIPY dopant. *Adv. Opt. Mater.* **8**, 2000483 (2020).
3. Fukagawa, H., Oono, T., Iwasaki, Y., Hatakeyama, T. & Shimizu, T. High-efficiency ultrapure green organic light-emitting diodes. *Mater. Chem. Front.* **2**, 704–709 (2018).
4. Uoyama, H., Goushi, K., Shizu, K., Nomura, H. & Adachi, C. Highly efficient organic light-emitting diodes from delayed fluorescence. *Nature* **492**, 234 (2012).
5. Wu, T.-L. *et al.* Diboron compound-based organic light-emitting diodes with high efficiency and reduced efficiency roll-off. *Nat. Photon.* **12**, 235–240 (2018).
6. Zhang, Y. *et al.* Multi-resonance induced thermally activated delayed fluorophores for narrowband green OLEDs. *Angew. Chem. Int. Ed.* **58**, 16912–16917 (2019).
7. Xu, Y. *et al.* Molecular-structure and device-configuration optimizations toward highly efficient green electroluminescence with narrowband emission and high color purity. *Adv. Opt. Mater.* **8**, 1902142 (2020).
8. Xu, Y. *et al.* Constructing charge-transfer excited states based on frontier molecular orbital engineering: Narrowband green electroluminescence with high color purity and efficiency. *Angew. Chem. Int. Ed.* **59**, 17442–17446 (2020).
9. Yang, M., Park, I. S. & Yasuda, T. Full-Color, narrowband, and high-efficiency

electroluminescence from boron and carbazole embedded polycyclic heteroaromatics. *J. Am. Chem. Soc.* **142**, 19468–19472 (2020).

10. Zhang, Y. *et al.* Achieving pure green electroluminescence with CIEy of 0.69 and EQE of 28.2% from an aza-fused multi-resonance emitter. *Angew. Chem. Int. Ed.* **59**, 17499–17503 (2020).
11. Ishigaki, Y., Shimajiri, T., Takeda, T., Katoono, R. & Suzuki, T. Longest C–C single bond among neutral hydrocarbons with a bond length beyond 1.8 Å. *Chem* **4**, 795–806 (2018)
12. Shi-Jie X. Huang-Rhys factor and its key role in the interpretation of some optical properties of solids. *Acta Phys. Sinica* **68**, 166301–11 (2019).
13. Heller, E. R. & Richardson, J. O. Semiclassical instanton formulation of Marcus–Levich–Jortner theory, *J. Chem. Phys.* **152**, 244117 (2020).
